# Phlebotomine sand flies (Psychodidae: Phlebotominae) in an area of canine infection caused by *Leishmania infantum* in the state of Amapá, eastern Amazon

**DOI:** 10.1590/S1984-29612023054

**Published:** 2023-09-08

**Authors:** Lourdes Marina Bezerra Pessoa, Engels Harmet Carvalho Pinto, Thiago Evangelista Silva Chaves, Gabriele da Silva Rabelo, Adrielly Lobato Brito, Volmir Miguel Zanini, Márcio Claudio de Lima Nunes, Lúcio André Viana

**Affiliations:** 1 Programa de Pós-graduação em Biodiversidade Tropical, Universidade Federal do Amapá – UNIFAP, Macapá, AP, Brasil; 2 Centro de Entomologia, Secretaria Municipal de Saúde do Município de Macapá, Macapá, AP, Brasil; 3 Laboratório de Estudos Morfofisiológicos e Parasitários, Departamento de Ciências Biológicas e da Saúde, Universidade Federal do Amapá – UNIFAP, Macapá, AP, Brasil; 4 Laboratório Central de Saúde Pública do Amapá, Macapá, AP, Brasil

**Keywords:** Sand fly, leishmaniasis, diversity, Amazon, Flebótomos, leishmaniose, diversidade, Amazônia

## Abstract

In 2017, the Brazilian State of Amapá registered the first occurrence of visceral leishmaniosis (VL) in 17 dogs in the outskirts of the capital, Macapá. Given the lack of knowledge on phlebotomines in that area, this study aimed to survey the fauna of these Diptera. Sampling was performed using CDC light traps placed at ten sampling sites. The specimens captured were *Evandromyia walkeri* (n=237), *Nyssomyia antunesi* (n=65) and *Bichromomyia flaviscutellata* (n=6). The phlebotomine species composition resulted in low species diversity, and none of the main vectors of the etiological agent of VL were identified in the study area.

## Resumo

Em 2017, o estado do Amapá registrou a primeira ocorrência de Leishmaniose Visceral (LV) em 17 cães, na periferia da capital, Macapá. Tendo em vista a falta de conhecimento sobre flebotomíneos nessa área, este trabalho teve como objetivo fazer um levantamento da fauna desses dípteros. A amostragem foi realizada usando-se armadilhas de luz CDC, colocadas em dez locais de amostragem. Os espécimes capturados foram *Evandromyia walkeri* (n=237), *Nyssomyia antunesi* (n=65) e *Bichromomyia flaviscutellata* (n=6). A composição de espécies de flebotomíneos resultou em baixa diversidade de espécies, e nenhum dos principais vetores do agente da LV foi identificado na área de estudo.

Phlebotomine sand flies (Diptera: Psychodidae) are important vectors of human pathogens that have a great impact on public health, of which Leishmaniasis caused by *Leishmania* species is the most important (Alvar et al., 2012). Leishmaniasis is a neglected tropical disease caused by the digenetic protozoan parasite of the genus *Leishmania. Leishmania* parasites cause a wide spectrum of clinical manifestations, which includes cutaneous (CL), mucosal (ML) and visceral leishmaniasis (VL) (OPS, 2019).

At least 20 Leishmania species are known to cause human disease, transmitted by different phlebotomine sand fly species (Cecílio et al., 2022). In Brazil, the mainly etiological agent of VL is *Leishmania infantum*, where transmission occurs through the bite of the infected phlebotomine sand fly species *Lutzomyia longipalpis*, while CL has different vectors and etiologic agents, the principal vectors being *Nyssomyia whitmani*, *Nyssomyia intermedia*, *Nyssomyia neivai*, *Migonemyia migonei* and *Psychodopygus wellcomei* (OPS, 2019; Lainson & Rangel, 2005).

The first case of VL in a domestic dog in the Macapá municipality was reported in 2017 (Brasil, 2018). Since then, VL has been detected in dogs in Macapá and Mazagão. Despite the occurrence of canine cases and the possible establishment of an urban VL transmission cycle, there is a lack of knowledge regarding sand fly vectors. Studies on sand fly fauna and their distribution in the Macapá municipality are lacking with only one published report available so far on the sand fly fauna in that region (Cavalcante et al., 2021). The purpose of this study was to investigate the phlebotomine sand fly fauna in areas of canine infection caused by *L. infantum* in Macapá municipality of the state of Amapá, Brazil.

This study was conducted in an urban area of Macapá, Amapá, Brazil (0° 2' 4” N, 51° 3' 60” W). The municipality has an estimated population of 474, 706 inhabitants. According to the Köppen climate classification, the region’s climate is equatorial, hot, and humid, with two distinct seasons: rainy season from December to July and dry season from August to November. The vegetation of this area comprises the Cerrado and lowland forests (Brasil, 2008). It is worth emphasizing that these environments are undergoing a rapid and disorderly process of urban occupation.

**Figure 1:**
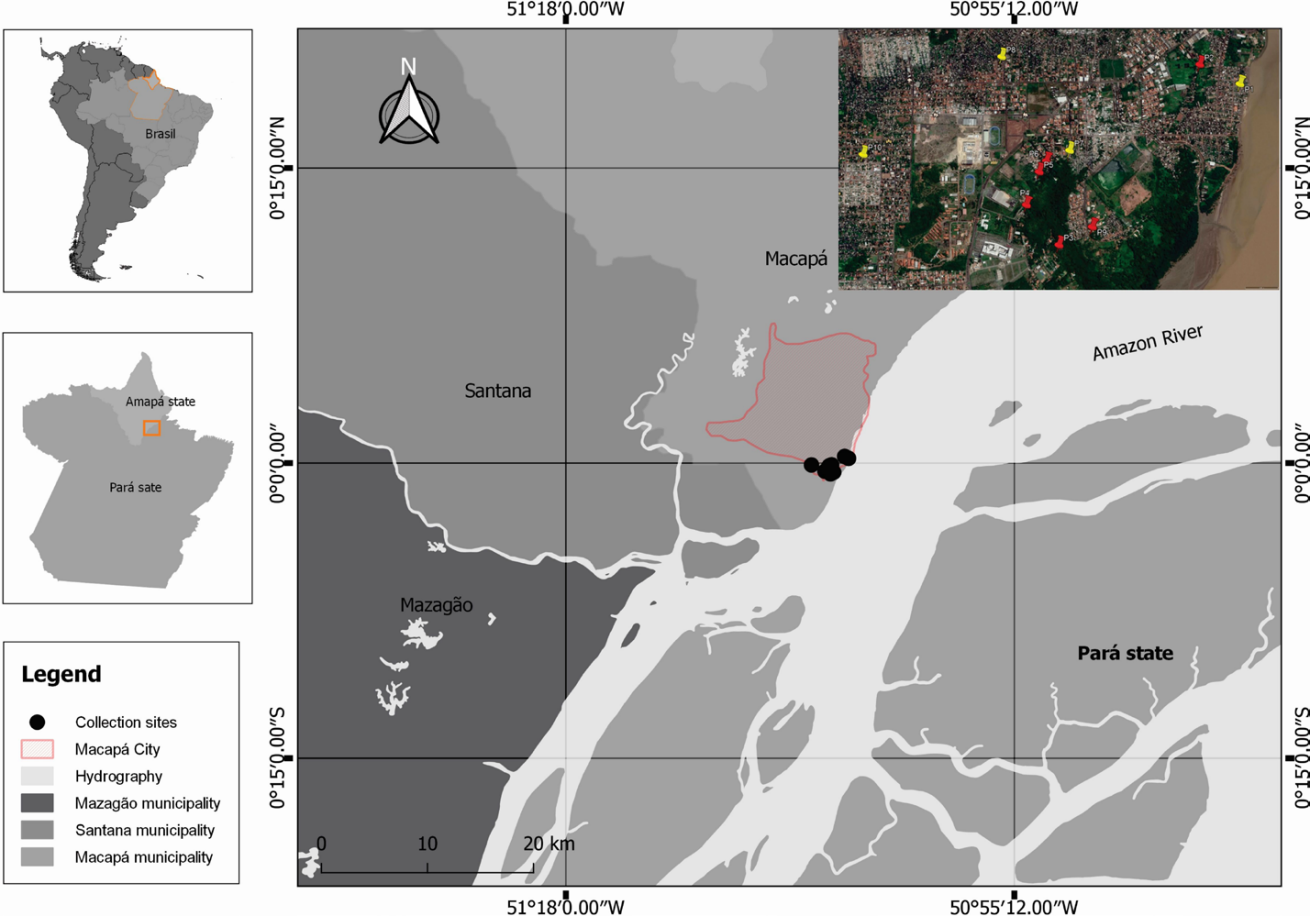
Sampling sites where phlebotomines were captured with CDC light traps in the urban area of Macapá, Amapá, Brazil. Red pins show phlebotomine-positive locations. Source: Google Earth. Nov/2018.

Sampling locations were selected based on presumed sites of Canine Visceral Leishmaniasis (CVL) infection (Brasil, 2018). Thus, ten residences were selected in the Araxá, Zerão, Pedrinhas, and Jardim Marco Zero neighborhoods (Figure 1). Entomological captures were performed on three consecutive nights each month, from December 2017 to November 2018, corresponding to 4,320 trap-nights in the study period. CDC light traps were placed at 6:00 p.m. and removed at 6:00 a.m. in the following day (from dusk to dawn). Traps were placed 1.5 m above the ground level in a peridomicile environment, preferably near shelters for domestic animals. After triaging, the sand flies were stored in 70% alcohol, diaphanized with 10% potassium hydroxide (KOH) solution, and clarified with lactophenol. Finally, the specimens were mounted on slides with coverslips using a drop of Berlese. The taxonomic criteria and nomenclature were followed based on the description by Galati (2018).

In total, 308 sand fly specimens were captured, of which 127 (41.2%) were females and 181 (58.8%) males. The insects belonged to three species. Identification of species revealed *Nyssomyia antunesi*, *Bichromomyia flaviscutellata* and *Evandromyia walkeri* (Table 1).

**Table 1:** Abundance of sand fly species captured in the Municipality of Macapá, Amapá, Brazil, from December 2017 to November 2018.

		**Species**			
**Month**	***Nyssomyia antunesi* N (**♀/♂**)**	***Bichromomyia flaviscutellata* N (**♀/♂**)**	***Evandromyia walkeri* N (**♀/♂**)**	**Total N (**♀/♂**)**	**%**
Dec/17	6 (3/3)	1 (0/1)	18 (11/7)	25 (14/11)	8.12
Jan/18	5 (3/2)	3 (3/0)	15 (8/7)	23 (14/9)	7.47
Feb/18	35 (21/14)	0	139 (41/98)	174 (62/112)	56.49
Mar/18	4 (2/2)	0	2 (0/2)	6 (2/4)	1.95
Apr/18	0	2 (0/2)	1 (0/1)	3 (0/3)	1.00
May/18	5 (4/1)	0	9 (6/3)	14 (10/4)	4.55
Jun/18	0	0	0	0	0.00
Jul/18	0	0	1(1/0	1 (1/0)	0.32
Aug/18	3 (3/0)	0	1(1/0)	4 (4/0)	1.00
Sep/18	6 (5/1)	0	17(6/11)	23 (11/12)	7.47
Oct/18	1 (1/0)	0	3(0/3)	4 (1/3)	1.30
Nov/18	0	0	31(8/23)	31(8/23)	10.10
**Total**	65 (42/23)	6 (3/3)	237 (82/155)	308 (127/181)	100
**%**	21.10	1.95	76.95		100

**Figure 2:**
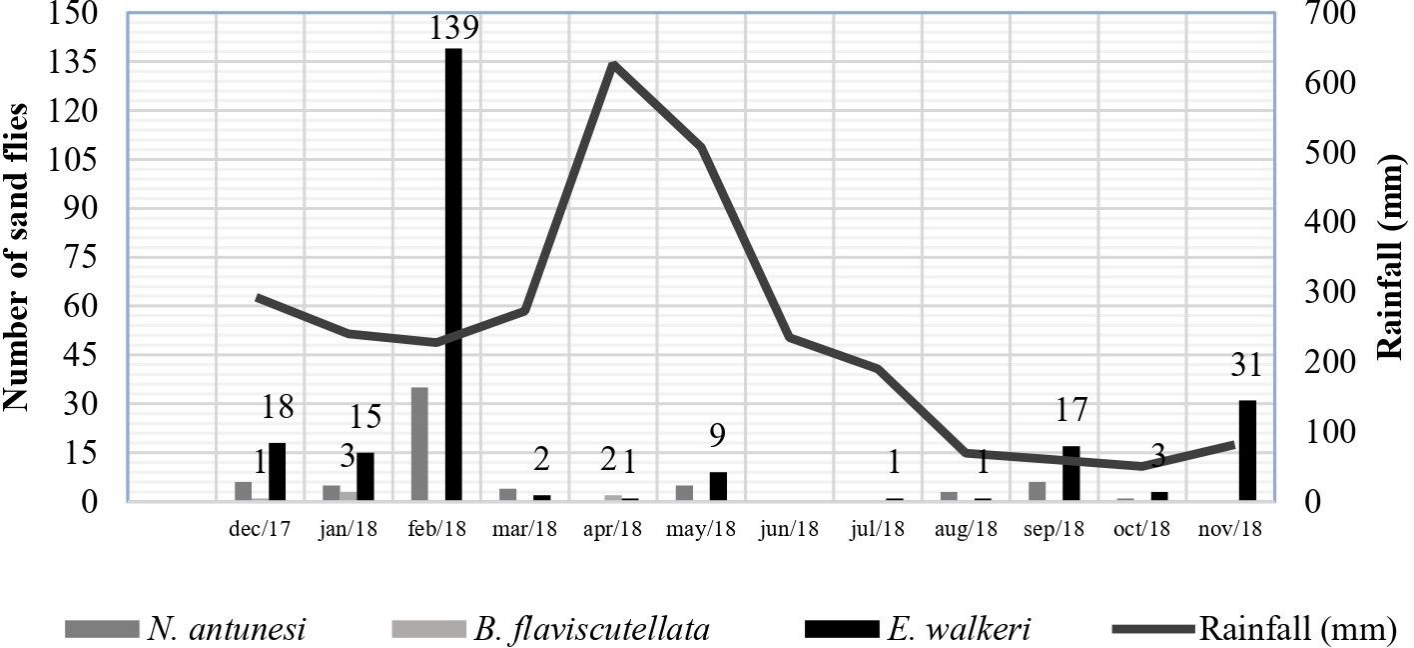
Number of captured sandflies accordingly to rainfall in Macapá, State of Amapá, from December 2017 to November 2018.

Among the ten capture sites, six (P2, P3, P4, P5, P6, and P9) were positive for phlebotomines (Figure 1), corresponding to the neighborhoods Marco Zero and Pedrinhas. The abundance and temporal fluctuations of sand flies can vary greatly from region to region (Guimarães et al., 2012). Vector distribution depends on environmental and biological factors like availability of hosts and weather conditions. In this study, the largest number of specimens was captured during the period of greatest precipitation, from December 2017 to July 2018 (245/308), corresponding to 80% of captures (Figure 2). Insect peaks were observed in the rainy seasons and in the months with less rain, such as November. In months with more cumulated rainfall, such as March, April and May, low densities of *Evandromyia walkeri* were found (Figure 2). On the other hand, February with the lowest accumulated precipitation represented the month with the highest density of this species together with *N. antunesi*. Thus, in this study, the highest number of specimens occurred in February corresponding to 56% (174/308) of the total number of sand flies captured in the state of Amapá, followed by November with 10% (31/308). None of the specimens were captured in June.

No association between rainfall and the abundance of sand flies was observed. These findings may be a result of the high rainfall fluctuations and difficulties in finding trends as there was no pattern. Although these meteorological variables may be related as predictor variables, they are not the only ones. Other variables may be determining factors in the densities of the sand flies in the area, such as microclimate, terrain, fauna, and vegetation.

Only one study has been conducted on the sand fly fauna in Macapá (Cavalcante et al., 2021). Our results corroborated with the same, in which there was little diversity of captured species and the largest number of specimens was captured during the period of greatest precipitation, with the males being more abundant than female specimens (Table 1). In this study, only three species were identified over the sampled year, indicating that species diversity was low, even though 77 phlebotomine species are known to occur in the State of Amapá (Galati, 2018).

The most abundant species found in this study was *E. walkeri*, contributing to 77% of the captured sand flies, mainly in the month of February in the rainy season. This species is widely distributed in both Central and South America and is often found in chicken coops (Young & Duncan, 1994). Although the epidemiological importance of *E. walker* is unknown, the species was recently found to be naturally infected with *L. braziliensis* DNA in Amazon region (De Ávila et al., 2018). The second-most abundant species, *N. antunesi* (21%) has tested positive for *Leishmania* DNA, suggesting its potential role as vector of *Leishmania* species (Pereira et al., 2019; Costa et al., 2021; Pimentel et al., 2022). In Central-Western Brazil, the pooled *N. antunesi* DNA sample was also found to be positive for *L. chagasi* (Thies et al., 2013). These facts emphasize the need for further investigation of this species in the context of local VL epidemiology. *Bichromomyia flaviscutellata* had the smallest number of captured specimens, which only occurred during the rainy season. It is rarely found in CDC light traps, as this species is considered highly zoophilic and predominates in forest areas, where it transmits *Leishmania amazonensis* at the soil level among rodents and marsupials (Ready et al., 1983). According to Rangel et al. (2018), *B. flaviscutellata* is a secondary vector of *L. infantum* among foxes, given that these canids are frequently found infected with *L. amazonensis*.

Although *L. longipalpis* was not found at the sampled sites in the present study, it was registered in the State of Amapá in 2013 in the municipality of Ferreira Gomes, 137 km from the state capital (Galardo et al., 2013). Therefore, this species may have been present in very small numbers, considering that the sampling effort undertaken in this study was unable to detect its presence. Another hypothesis is that the transmission of the VL agent involves alternative vectors, such as *N. antunesi* and/or *B. flaviscutellata* which, according to literature studies, has already been associated with *L. chagasi* life cycle under different circumstances.

Taken together, the phlebotomine entomofauna survey revealed low species diversity, along with the absence of the classical vector of *L. chagasi* the etiological agent of CVL in the study area. The registration of sand fly species as potential vectors of *Leishmania* in the Amazon region, such as *B. flaviscutelata* and *E. walkeri*, becomes a relevant factor in the transmission of *Leishmania* parasites in anthropic environments. With this, further efforts are required for better assessment of the possible epidemiological importance of the species captured in the surveyed area, with the main focus on *Leishmania* spp. detection.
